# IL-4Rα-Dependent Alternative Activation of Macrophages Is Not Decisive for *Mycobacterium tuberculosis* Pathology and Bacterial Burden in Mice

**DOI:** 10.1371/journal.pone.0121070

**Published:** 2015-03-19

**Authors:** Reto Guler, Suraj P. Parihar, Suzana Savvi, Erin Logan, Anita Schwegmann, Sugata Roy, Natalie E. Nieuwenhuizen, Mumin Ozturk, Sebastian Schmeier, Harukazu Suzuki, Frank Brombacher

**Affiliations:** 1 International Centre for Genetic Engineering & Biotechnology (ICGEB), Cape Town component and Institute of Infectious Diseases and Molecular Medicine (IDM), Division of Immunology, University of Cape Town, Cape Town, South Africa; 2 Massey University, Institute of Natural and Mathematical Sciences, Auckland, New Zealand; 3 RIKEN Center for Life Science Technologies, Division of Genomic Technologies, Yokohama, Japan; Karolinska Institutet, SWEDEN

## Abstract

Classical activation of macrophages (caMph or M1) is crucial for host protection against *Mycobacterium tuberculosis* (*Mtb*) infection. Evidence suggests that IL-4/IL-13 alternatively activated macrophages (aaMph or M2) are exploited by *Mtb* to divert microbicidal functions of caMph. To define the functions of M2 macrophages during tuberculosis (TB), we infected mice deficient for IL-4 receptor α on macrophages (LysM^cre^IL-4Rα^-/lox^) with *Mtb*. We show that absence of IL-4Rα on macrophages does not play a major role during infection with *Mtb* H37Rv, or the clinical Beijing strain HN878. This was demonstrated by similar mortality, bacterial burden, histopathology and T cell proliferation between infected wild-type (WT) and LysM^cre^IL-4Rα^-/lox^ mice. Interestingly, we observed no differences in the lung expression of inducible nitric oxide synthase (iNOS) and Arginase 1 (Arg1), well-established markers for M1/M2 macrophages among the *Mtb*-infected groups. Kinetic expression studies of IL-4/IL-13 activated bone marrow-derived macrophages (BMDM) infected with HN878, followed by gene set enrichment analysis, revealed that the MyD88 and IL-6, IL-10, G-CSF pathways are significantly enriched, but not the IL-4Rα driven pathway. Together, these results suggest that IL-4Rα-macrophages do not play a central role in TB disease progression.

## Introduction

Classical activation of macrophages results in concomitant production of reactive nitrogen intermediates (RNIs) such as nitric oxide (NO), killing intracellular *Mtb* [1]. In contrast, alternative activation of macrophages is induced by IL-4 or IL-13 via the IL-4 receptor-alpha chain (IL-4Rα) and results in decreased NO production by induction of Arg1, which competes with iNOS for the common substrate L-Arginine [2, 3]. Considering the hostile milieu inside caMph, *Mtb* uses evasion mechanisms and might potentially subvert the transcriptional network to hide in alternatively activated macrophages, thereby avoiding caMph effector killing functions. Indeed, *Mtb* has been shown to interfere with polarization of caMph by restricting MyD88-dependent TLR signalling through the secretion of the virulence factor ESAT-6 [4]. Elevated levels of IL-4 was found in patients with progressive pulmonary TB and is related to the presence of pulmonary cavities [5–9]. *In vitro* infection of PBMC with clinical Beijing strain HN878 resulted in the upregulation of IL-4/IL-13 [10]. Earlier studies reported that T_H_2 responses and aaMph have been implicated in the re-activation of latent TB in mice [11] and intracellular persistence of *Mtb* in murine macrophages, [12] respectively. Moreover, Arg1 was induced in HN878-infected murine macrophages [13] and is expressed in lung granulomas of TB patients [14] suggesting possible avoidance of NO mediated killing by the hypervirulent strain of *Mtb*.

The induction of Arg1 by T_H_2 cytokines in macrophages is well studied and requires STAT6 signalling through the IL-4Rα chain [2, 15, 16]. However, we have limited information on the induction of Arg1 following mycobacterial infections. Two *in vitro* studies showed that *M*. *bovis* BCG induces Arg1 through a MyD88-dependent pathway but in an STAT6-independent manner [13, 17]. Qualls *et al*. characterized the pathway involved and revealed that *M*. *bovis* BCG-infected macrophages secrete MyD88-dependent IL-6, IL-10 and G-CSF that induces Arg1 expression in an autocrine/paracrine manner [13].

Here we expand on the *M*. *bovis* BCG *in vitro* studies by Qualls *et al*. and El Kasmi *et al*. [13, 17] and confirm their findings *in vivo* using the hypervirulent Beijing strain, *Mtb* HN878 and LysM^cre^IL-4Rα^-/lox^ mice, which are deficient for IL-4Rα in macrophages and neutrophils [18]. Even though neutrophils play important roles in the host response to acute tuberculosis, IL-4Rα responsive neutrophils seem not to have essential function in TB pathology [19, 20].

We show that the absence of IL-4Rα responsiveness on macrophages only marginally influenced acute bacterial burden, chronic pulmonary pathology and does not influence survival following infection with virulent and hypervirulent strains of *Mtb*. Taken together, this suggests that IL-4Rα-activated macrophages are not required for TB disease progression since *Mtb* induces Arg1 production independent of the IL-4Rα signalling pathway.

## Materials and Methods

### Mice

Wild-type BALB/c, control littermates (IL-4Rα^-/lox^), IL-4Rα^-/-^ and macrophage cell-specific IL-4Rα deficient mice (LysM^cre^IL-4Rα^-/lox^) on a BALB/c background (8–12 weeks) were kept under specific-pathogen-free conditions in individually ventilated cages.

### Ethics Statement

All experiments were performed in accordance with the South African National Standard (SANS 10386:2008) and University of Cape Town of practice for laboratory animal procedures. The protocol (Permit Number: 012/036) was approved by the Animal Ethics Committee, Faculty of Health Sciences, University of Cape Town, Cape Town, South Africa. All animal users had successfully completed the mandatory University of Cape Town animal handling courses and accredited by South African Veterinary Council. All procedures were performed under halothane anaesthesia.

### *Mtb* infection in mice

Mice were infected with *Mycobacterium tuberculosis* H37Rv and HN878 via the intranasal or aerosol route as described previously [21]. Body weight of *Mtb*-infected mice was measured twice a week and mice were monitored daily. If infected mice lost more than 20% of their original body weight or showed severe signs of illness, such as hunched up posture, coat staring, immobility and general lack of grooming, mice were considered moribund and were euthanized to minimize suffering. Lung weight index and bacterial loads in lungs and spleen of *Mtb*-infected mice were determined at different time points PI (post infection) as previously described [21, 22].

### Histopathology and immunohistochemistry

Lungs of *Mtb*-infected mice were fixed with 4% phosphate-buffered formalin, and 3 μm-thick sections were stained with either H&E or rabbit anti-mouse Ab to iNOS or goat anti-mouse Ab to Arg1 (Abcam). Detection was performed using HRP-labelled anti-rabbit and anti-goat Abs (Dako) respectively. The lung histopathology scores were graded from 0–10 based on perivascular/ peribronchiolar lymphocytic infiltrates, reduced ventilated alveolar spaces and extensive pulmonary lesions.

### Analysis of immune cell populations and IL-4Rα expression in lungs by FACS

Single cell suspensions of the lungs were stained as previously described [23–25]. Briefly, lung cells were subjected to staining with rat anti-mouse IL-4Rα PE (BD Biosciences) and rat anti-mouse antibodies for various markers of T cells/B cells/Mph/DCs/Neutrophils & Eosinophils (BD Biosciences) with blocking 1% rat serum and 1% anti-FcyRII/III. Cells were acquired using FACS Calibur (BD Biosciences) and analysed by FlowJo (TreeStar).

### Co-culture

CD4^+^ T cells from wild type (BALB/c) naive splenocytes (>94% purity) were sorted using MACS beads (Miltenyi Biotec) and labelled with Oregon green (Invitrogen), then cultured with CD11c-sorted macrophages isolated from the lung tissue of naïve non-infected or *Mtb*-infected mice. Co-cultured cells were collected after 4 days for CD4^+^ T cell staining and analysed for T cell proliferation using flow cytometry.

### Generation of BMDM and *Mtb in vitro* infection

BMDM were generated as previously described [26]. 5x10^6^ BMDM/well were treated with or without: 100 U/ml IL-4 and 100 U/ml IL-13 (BD Biosciences). After 24 hours, cells were washed twice with antibiotic-free media and the BMDM were infected in antibiotic-free medium with live logarithmic phase *Mtb* HN878 at a MOI 5:1 (bacilli:macrophage) in the presence or absence of activators. After 4 h of infection, BMDM were washed once with culture media and incubated with plus/minus activators, 10 μg/ml of gentamicin, 100 U/ml penicillin G and 100 μg/ml streptomycin. At 4, 12 and 48h PI, BMDM were lyzed with 1 ml of Qiazol and total RNA was extracted by miRNAeasy kit (Qiagen).

### Microarray

Total RNA (500 ng) was amplified using the Ambion total RNA amplification kit (Ambion) and was hybridized to Illumina mouse Sentrix bead chips WG-6V2 array (Illumina). Scanning of the chip was performed using Illumina BeadScan and data was generated using BeadStudio software packages (version 1.6). Two biological replicates were analysed and the data was deposited in the GEO database (GSE56736).

### Gene Set Enrichment Analysis

Genes were extracted from both Pathway Commons database [27] and National Cancer Institute Pathway Interaction Database [28]. The GSEA v2.0.13 tool [29] was used to conduct enrichment analysis. Multiple probes corresponding to the same gene were first collapsed by taking mean value of each probe set. Genes were then pre-ranked by using the metric score of log2 fold-changes. Normalized enrichment scores (NES), nominal p-values and false discovery rates (FDR) were computed by permuting sample labels 1000 times as previously described [29]. GenePattern [30] software was used for the generation of heatmaps of leading edge genes.

### Statistics

Data is represented as mean values ± SEM. Statistical analysis was performed using Student’s *t* test, two-tailed, unequal variance, defining differences to control groups as significant (*, *P* < 0.05; **, *P* < 0.01; ***, *P* < 0.001).

## Results

### IL-4Rα responsive macrophages contribute to containing early bacterial burden and chronic pulmonary inflammation

**Fig 1:**
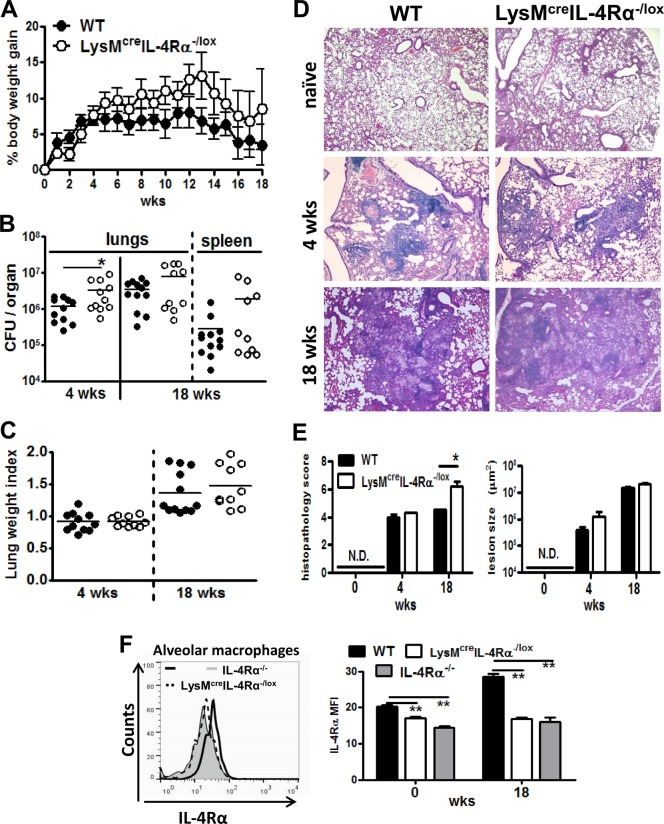
Increased acute bacterial burden and chronic pulmonary pathology in absence of IL-4Rα responsive macrophages following low-dose *Mtb* H37Rv infection . Wild-type (BALB/c), IL-4Rα^-/-^ and IL-4Rα macrophage cell-specific deficient mice (LysM^cre^IL-4Rα^-/lox^) were infected with *Mtb* H37Rv (100 CFU/mouse) by aerosol (n = 12–13 mice/group). (A) Percentages in body weight change are shown. (B) Mice were sacrificed at 4 and 18 weeks PI to determine bacterial loads in the lungs and spleen. (C) Lung weight indexes are shown. (D) At 0, 4 and 18 weeks PI, formalin-fixed lung sections were stained with H&E. Original magnification: 40X. (E) Lung sections of 5 mice per group were evaluated for pulmonary histopathology scores and quantification of total lesion sizes. N.D. = not detectable. (F) IL-4Rα expression was measured by flow cytometry on alveolar macrophages (SiglecF^+^CD11c^+^) at 0 and 18 weeks PI (*P < 0.05, **P < 0.01). Data shown are representative (A, D, E and F) and pooled (B, C) from two independent experiments.

Wild-type (BALB/c), IL-4Rα^-/-^ and IL-4Rα macrophage cell-specific deficient mice (LysM^cre^IL-4Rα^-/lox^) were infected via pulmonary aerosol route with 100 CFU/mouse of *Mtb*, H37Rv. All mice gradually increased in weight (Fig. 1A). The bacillary burden at 4 weeks after infection was significantly increased in macrophage cell-specific IL-4Rα deficient mice when compared to wild-type mice (Fig. 1B). However, in terms of biological significance this CFU increase was only marginal with a ½ log increase. In contrast, no significant difference was observed in bacillary burden in the lungs and spleens between all the groups at 18 weeks PI. The lung weight index, a surrogate indicator of inflammation, did not reveal any differences between the groups during the infection (Fig. 1C). At 4 and 18 weeks PI all mouse groups displayed similar well-defined granuloma formation (Fig. 1D). However, the histopathology score, as described in the Materials and Methods, at 18 weeks PI revealed significantly increased inflammation in macrophage cell-specific IL-4Rα deficient mice, when compared to wild-type BALB/c mice (Fig. 1E). Pulmonary histopathology, bacterial burden, iNOS and Arg1 expression were similar between wild-type BALB/c and IL-4Rα^-/-^ mice (S1 Fig.). These results suggest that IL-4Rα responsive macrophages contribute to containing bacterial growth during the acute phase and down-modulate inflammation during the chronic phase of *Mtb* infection.

### Reduced IL-4Rα expression on alveolar macrophages in LysM^cre^IL-4Rα^-/lox^ mice following *Mtb* infection

We previously showed that macrophage-specific IL-4Rα expression was impaired in OVA-challenged lungs, [23] and in mesenteric lymph nodes [18] and liver granulomas [31] from *S*. *mansoni* infected LysM^cre^IL-4Rα^-/lox^ mice. Here we characterized the IL-4Rα expression by flow cytometry in the lungs during low dose H37Rv infection (Fig. 1F). The results showed that the IL-4Rα expression is genetically abrogated in alveolar macrophages in LysM^cre^IL-4Rα^-/lox^ when compared to BALB/c wild-type mice at 18 weeks PI.

### IL-4Rα deficient macrophages show similar iNOS and Arg1 expression during low dose *Mtb* infection

**Fig 2:**
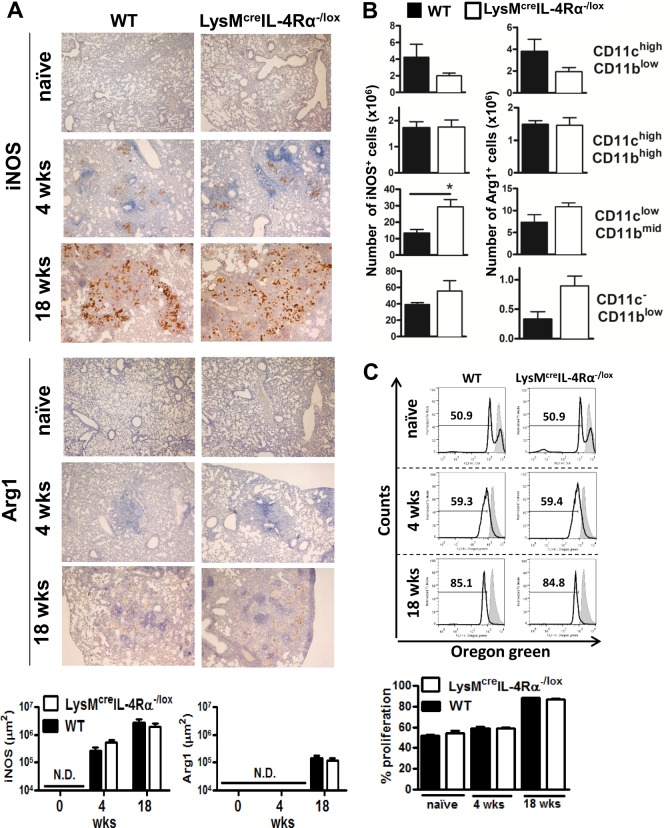
No major differences in expression of iNOS, Arg1, lung immune cell populations and T cell proliferation between wild-type and macrophage cell-specific IL-4Rα deficient mice following low-dose *Mtb* H37Rv infection (100 CFU/mouse). (A) iNOS and Arg1 staining (brown colour) from lung sections collected at indicated times PI, original magnification: 40X. Lung sections from 5 mice/group were quantified. N.D. = not detectable. (B) iNOS and Arg1 expression on various immune cells were analysed by flow cytometry at 18 weeks PI (6–7 mice/group, *P < 0.05). (C) T cell proliferation with co-cultured CD11c-sorted macrophages from the lung tissue of naïve non-infected and mice infected with H37Rv (100 CFU/mouse) by aerosol at 4 and 18 weeks. Data shown in A is representative of two independent experiments and results obtained in B and C are from one experiment.

Numerous studies in allergic airway and parasitic diseases have reported that the classical caMph marker iNOS is increased and the typical aaMph marker Arg1 is decreased in macrophage cell-specific IL-4Rα deficient mice when compared to control groups [18, 23, 31, 32]. In contrast, our immunohistochemistry results show that LysM^cre^IL-4Rα^-/lox^ mice had comparable levels of iNOS and Arg1 (Fig. 2A) when compared to wild-type mice during acute/chronic phase of infection with low-dose *Mtb* H37Rv (100 CFU/mouse). Interestingly, Arg1 was not yet expressed in the lungs of mice at the 4 week time point, suggesting that induction occurs later during the course of disease. Finally, iNOS and Arg1 expression (Fig. 2B) was further confirmed at 18 weeks PI by flow cytometry in alveolar macrophages (CD11c^high^CD11b^low^), conventional dendritic cells (CD11c^high^CD11b^high^), recruited interstitial macrophages (CD11c^low^CD11b^mid^) and monocytes (CD11c^-^CD11b^low^) using cell surface markers as previously defined [24, 25]. Gating strategy is shown in S2 Fig. Together, these data demonstrate that Arg1 is induced through an IL-4Rα-independent pathway and is only expressed after a well-established *Mycobacterium* infection in macrophage cell-specific IL-4Rα deficient mice.

### Similar T cell proliferation in the absence of IL-4Rα-dependent macrophages during low dose *Mtb* infection

It has been reported that IL-4 exposed macrophages suppress proliferation of T cells via a STAT6-dependent pathway [33]. We co-cultured Oregon Green-labelled CD4^+^ T cells with macrophages isolated from naïve and *Mtb*-infected LysM^cre^IL-4Rα^-/lox^ mice and their corresponding wild-type mice (Fig. 2C). Absence of IL-4Rα responsive macrophages did not influence the proliferative activity on T cells. In conclusion, these results indicate that the IL-4Rα expression on macrophages is not required to alter cellular lung composition and T cell proliferation during *Mtb* infection.

### Deletion of IL-4Rα on macrophages does not influence survival or pathology after high dose *Mtb* H37RV infection

**Fig 3:**
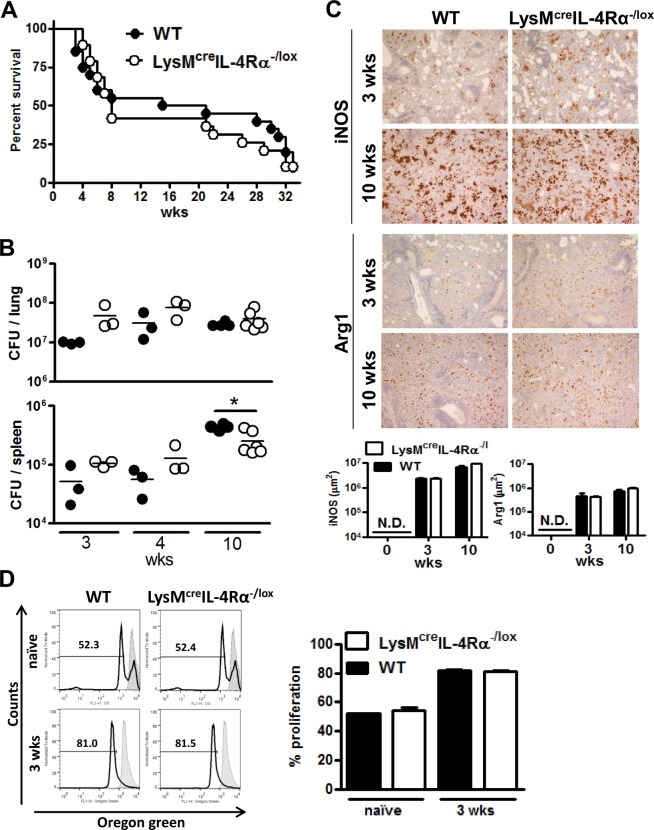
Similar mortality, inflammation, bacterial burden, Arg1/iNOS expression and T cell proliferation in wild-type and LysM^cre^IL-4Rα^-/lox^ mice following high dose infection with *Mtb* H37Rv . Wild-type (BALB/c) and IL-4Rα macrophage cell-specific deficient mice (LysM^cre^IL-4Rα^-/lox^) were infected intranasally with high dose of 10^4^ CFU/mouse of *Mtb* H37Rv (n = 20/group). (A) Survival of infected mice was recorded weekly. (B) Individual bacterial titers (CFU/organ) with group medians are shown (*P < 0.05). (C) iNOS and Arg1 staining (brown colour) from lung sections collected at 3 and 10 weeks PI. Lung sections from 5 mice/group were quantified. Original magnification: 40X. N.D. = not detectable. (D) T cell proliferation with co-cultured CD11c-sorted macrophages from the lung tissue of naïve non-infected and 3 weeks infected mice. All data shown is representative of two independent experiments except for B which is representative of three independent experiments.

To determine the absence of macrophage IL-4Rα signalling on the susceptibility to high dose *Mtb* infection, wild-type (BALB/c) and IL-4Rα macrophage cell-specific deficient mice (LysM^cre^IL-4Rα^-/lox^) were infected intranasally with 10^4^ CFU/mouse of H37Rv (Fig. 3A). A high infectious dose resulted in rapid death within 8 weeks of infection and the remaining mice progressively lost weight and died within the 8 months of the monitoring period. Kaplan-Meier analysis did not reveal any significant difference (*P* = 0.729) in survival between both groups of mice. Lung bacterial burden and *Mtb* dissemination to the spleen (Fig. 3B), iNOS and Arg1 expression (Fig. 3C) and T cell proliferation (Fig. 3D) from *Mtb*-infected LysM^cre^IL-4Rα^-/lox^ and BALB/c mice were similar. Taken together, these data demonstrate that the absence of IL-4Rα-dependent macrophages did not influence the survival and immune response to high dose infection with H37Rv.

### Deletion of IL-4Rα on macrophages does not influence survival and innate immunity to a hypervirulent strain of *Mtb* (HN878)

**Fig 4:**
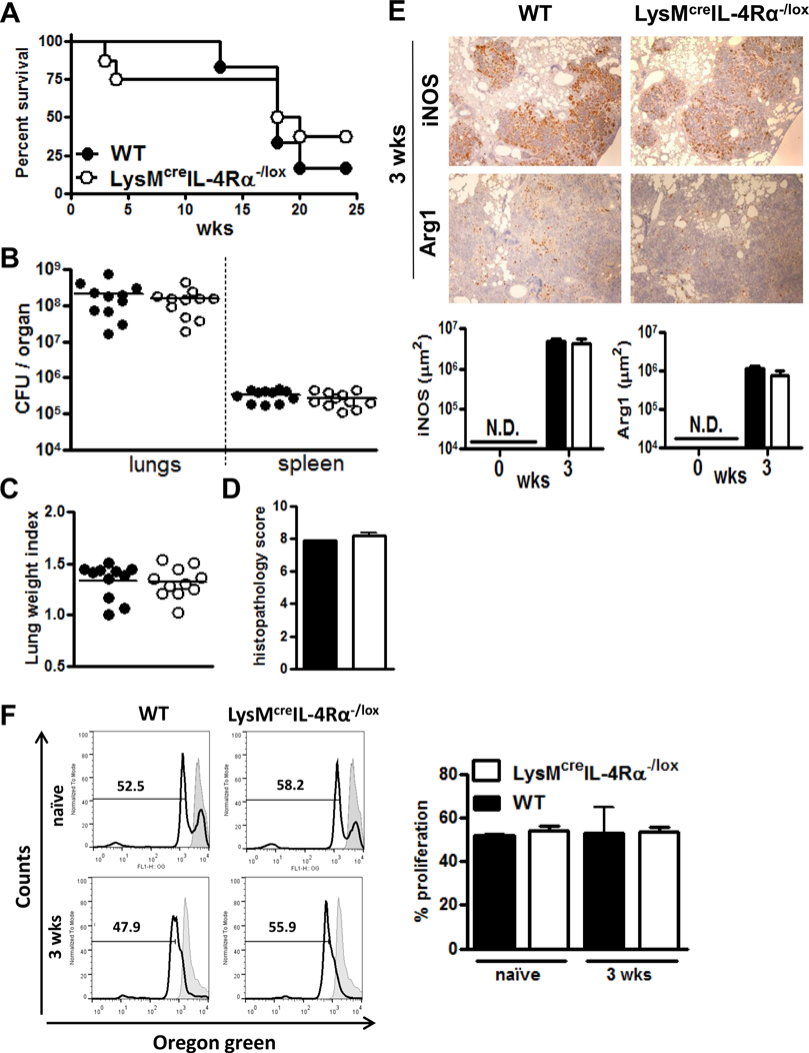
Macrophage cell-specific IL-4Rα deficient and wild-type mice displayed similar mortality, inflammation and T cell proliferation to hypervirulent *Mtb* HN878 infection . Wild-type (BALB/c) and IL-4Rα macrophage cell-specific deficient mice (LysM^cre^IL-4Rα^-/lox^) were infected intranasally with 500 CFU/mouse of hypervirulent *Mtb* HN878. (A) Survival of infected mice was recorded weekly (n = 6–8/group). (B) Individual bacterial titers (CFU/organ), (C) lung weight indexes and (D) histopathology score with group medians are shown at 3 weeks PI (n = 11/group). (E) iNOS and Arg1 staining (brown colour) from lung sections collected at 3 weeks PI. Lung sections from 5 mice/group were quantified. Original magnification: 40X. N.D. = not detectable. (F) T cell proliferation with co-cultured CD11c-sorted macrophages from the lung tissue of naïve non-infected and mice infected with HN878 at 3 weeks PI. Data shown in A-E is representative of two independent experiments and results obtained in F is from one experiment.

To assess whether IL-4Rα responsive macrophages play a role in the susceptibility to a hypervirulent strain of *Mtb*, LysM^cre^IL-4Rα^-/lox^ and wild-type BALB/c mice were infected with clinical isolate strain HN878 of *Mtb*. Here we show that LysM^cre^IL-4Rα^-/lox^ had similar high mortality rates (Fig. 4A) and mycobacterial burden (Fig. 4B) in the lungs and spleen when compared to BALB/c wild-type mice. Kaplan-Meier analysis did not reveal any significant difference (*P* = 0.621) in survival between both groups of mice. Lung weight index and histopathology scores of H&E stained lung sections did not reveal any significant differences among the HN878-infected groups (Fig. 4C and 4D). iNOS and Arg1 expression were similar between both groups of mice when analysed at 3 weeks PI (Fig. 4E). Moreover, T cell proliferation was not altered in the presence of macrophages isolated from the different infected groups (Fig. 4F). Together, these results illustrated that IL-4Rα responsive macrophages do not influence survival and innate protective immunity against the hypervirulent strain HN878 of *Mtb*.

### The MyD88 but not IL-4Rα dependent pathway is enriched in HN878-infected macrophages

**Fig 5:**
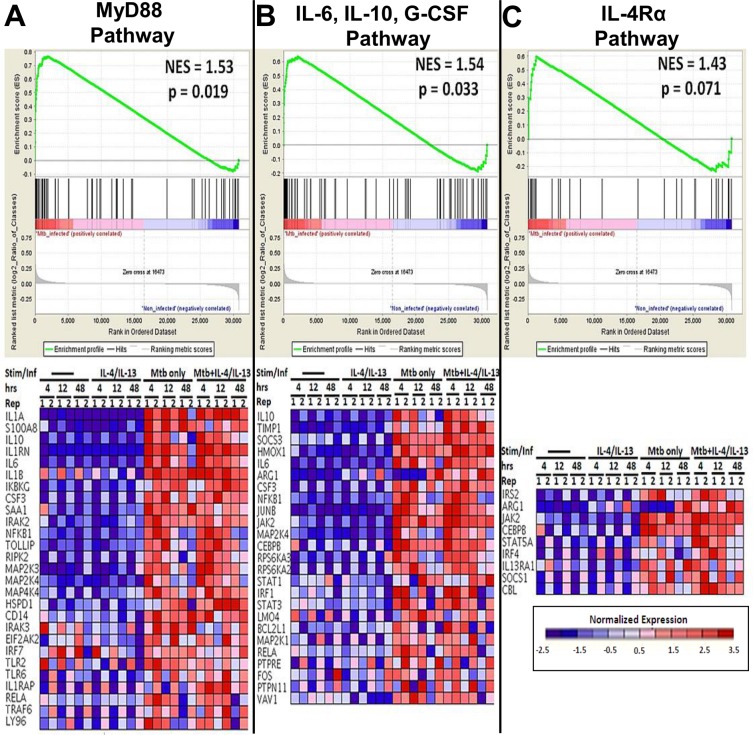
MyD88 and IL-6, IL-10, G-CSF-dependent pathway genes are significantly enriched in HN878 infected vs. non-infected macrophages . BMDM were stimulated with IL-4/IL-13 or left untreated. After 24 hours of stimulation, cells were infected with HN878. Total RNA was extracted at 4, 12 and 48 hours PI for microarray and GSEA analysis. Enrichment plots and heat maps for (A) MyD88, (B) IL-6, IL-10, G-CSF and (C) IL-4Rα pathway are shown. Enrichment analysis compared log2-fold changes in *Mtb*-infected samples vs. non-infected samples. The rows in heat maps are listed according to pre-ranking metric scores. Replicates shown are from two independent experiments.

We employed gene set enrichment analysis (GSEA) on genome-wide gene expression data to determine whether IL-4Rα signalling pathway genes or MyD88-mediated pathway genes associated with Arg1 expression were enriched during *Mtb* infection. Expression profiles of BMDM infected with HN878 *Mtb* were analysed, and GSEA was performed with gene sets for IL-6, IL-10 and G-CSF (CSF3) pathways, MyD88-mediated pathways and the IL-4Rα pathway. GSEA revealed that genes from the MyD88-mediated pathways (*P* = 0.019) and IL-6, IL-10 and G-CSF cytokine pathways (*P* = 0.033) are highly enriched during HN878 infection when compared to non-infected samples (Fig. 5A and 5B). However, genes in the IL-4Rα pathway are not significantly enriched during *Mtb* infection when compared to non-infected samples with a *p*-value of 0.07 (Fig. 5C). Collectively, these results indicate that Arg1 may be induced by a MyD88-dependent cell extrinsic pathway rather than an IL-4Rα dependent pathway during *Mtb* infection by the hypervirulent strain HN878.

## Discussion

AaMph are activated by IL-4/IL-13 to induce Arg1, subverting the host NO-based mycobactericidal activity. This suggests that induction of Arg1 is an important evasion tactic exploited by *Mtb* to thrive inside caMph. Indeed, specific depletion of Arg1 in macrophages using the Cre-LoxP recombination system resulted in elevated NO levels that contributed to 1-log reduction in lung colony counts following *Mtb* H37Rv infection [17]. In aaMph, Arg1 is induced by IL-4/IL-13 via the IL-4Rα chain. Thus, deleting the IL-4Rα signalling pathway would lead to increased availability of L-Arginine substrate for iNOS, leading to enhanced NO-mediated killing functions. Indeed, using the Cre-LoxP system, we have previously shown that when IL-4Rα is specifically deleted in macrophage cells, Arg1 activity is reduced and NO production is increased. This leads to enhanced killing of *L*. *major* parasites [32] and results in protective immunity against *N*. *brasiliensis* [18]. Previously, it was not known whether the absence of IL-4Rα during mycobacterial infection influences Arg1 and NO expression, and may therefore alter susceptibility to *Mtb*. Therefore, we investigated the role of IL-4Rα-activated alternative macrophages during *Mtb* infection using LysM^cre^IL-4Rα^-/lox^ mice, where the IL-4Rα allele is flanked by loxP sites and the Cre recombinase is under the control of the lysozyme M gene (LysM^cre^), thus restricting Cre-mediated excision of IL-4Rα in macrophages and neutrophils. Although the deletion efficiency of IL-4Rα in mature macrophages is not 100%, [34] we observed similar IL-4Rα deletion between LysM^cre^IL-4Rα^-/lox^ and IL-4Rα^-/-^ mice during *Mtb* infection (Fig. 1F). Previously we found that Th1/Th2 polarization and lymphocyte proliferation was not altered in LysM^cre^IL-4Rα^-/lox^ when compared to WT BALB/c mice [18].

Interestingly, we found similarly high induction of both iNOS and Arg1 in IL-4Rα^-/-^ (S1 Fig.), LysM^cre^IL-4Rα^-/lox^ and WT BALB/c mice. Expression of iNOS and Arg1 was consistently detected during the acute and chronic phases of infection with low and high doses of the virulent laboratory strain H37Rv, and also using the hypervirulent clinical isolate HN878. These results suggest that Arg1 expression in macrophages is induced via an IL-4Rα-independent pathway. Moreover, the absence of IL-4Rα on macrophages did not influence the susceptibility, mortality and pathology to virulent H37Rv, clinical CDC1551 (data not shown), and HN878 *Mtb* strains. In addition, lung bacterial burdens, *Mtb* (H37Rv) dissemination to spleen, and histopathology were similar between IL-4Rα^-/-^ (S1 Fig.), LysM^cre^IL-4Rα^-/lox^ and wild-type BALB/c mice. However, we did observe a significant increase in early lung bacterial loads and chronic histopathology scores in LysM^cre^IL-4Rα^-/lox^ when compared to wild-type BALB/c mice following low dose infection with H37Rv, although the differences were marginal. Bacterial burden, susceptibility, histopathology, Arg1 and iNOS expression and were similar between control littermates (IL-4Rα^-/lox^) and wild-type BALB/c mice infected with *Mtb* H37Rv and HN878 (data not shown).

Huber *et al*. reported that IL-4-exposed macrophages suppress T cell proliferation in a STAT6-dependent manner [33]. In contrast to Huber *et al*., our results revealed that T cell proliferation was not affected by the presence or absence of IL-4Rα on macrophages during *Mtb* infection with both virulent and hypervirulent strains. These differences could be attributed to IL-4Rα-independent production of Arg1 which is sufficient to suppress T cell proliferation in aaMph [35]. Similarly to our study, Arg1^+^ alveolar macrophages were observed in LysM^cre^IL-4Rα^-/lox^ mice during a pulmonary infection with *C*. *neoformans* [36]. The fact that in our hands LysM^cre^IL-4Rα^-/lox^ mice produced an equal amount of Arg1 when compared to wild-type BALB/c mice suggests that *Mtb* is responsible for driving Arg1 expression independently of the IL-4Rα signalling pathway. Indeed, Qualls *et al*. and El Kasmi *et al*. found *in vitro* that *M*. *bovis* BCG induces the expression of Arg1 in a manner that is dependent on MyD88 [13, 17]. This was demonstrated by the fact that *M*. *bovis* BCG infected MyD88 deficient macrophages, which displayed a lack of Arg1 expression, whereas Arg1 was induced in STAT6^-/-^ macrophages. Our study expands on their observations, as we show that in macrophages infected with the hypervirulent clinical isolate HN878 strain of *Mtb*, genes in the MyD88-dependent pathway are enriched for high expression as compared to the genes in the IL-4Rα-dependent pathway. We also found that the IL-6, IL-10 and G-CSF pathways are enriched during HN878 infection, in accordance with the findings of Qualls *et al*., who reported that these cytokines can act in an autocrine-paracrine manner to induce Arg1 via STAT3 phosphorylation during *M*. *bovis* BCG infection [13].

In addition, we investigated the *in vivo* involvement of IL-4Rα in the production of Arg1. We found that IL-4Rα-dependent alternative activation of macrophages is not decisive in the control of susceptibility and pathology to both virulent and hypervirulent strains of *Mtb*. Importantly, *Mtb* induces Arg1 but in an IL-4Rα-independent manner. We further show that virulent and clinical *Mtb* strains can induce Arg1 expression, potentially thriving in aaMph to establish persistence and survival.

## Supporting Information

S1 Fig No major differences in pulmonary histopathology, iNOS, Arg1 and bacterial burden between wild-type and IL-4Rα deficient mice following low-dose *Mtb*, H37Rv infection (100 CFU/mouse). (A) H&E, iNOS and Arg1 staining from lung sections collected at 0 and 18 wks post infection, original magnification: 40X. (B) Histopathology score, quantification of lesion sizes, iNOS and Arg1 quantification. N.D. = not detectable. (C) Lung weight indexes and bacterial burden in the lungs and spleen are shown (5 mice/group, *P < 0.05). All data shown is representative of two independent experiments.
(TIF)

S2 Fig Representative gating strategy with forward and side scatter to eliminate debris. CD11b and CD11c-expressing subsets in the lung were defined as R1: conventional dendritic cells (CD11c^high^CD11b^high^), R2: alveolar macrophages (CD11c^high^CD11b^low^), R3: recruited interstitial macrophages (CD11c^low^CD11b^mid^) and R4: monocytes (CD11c^-^CD11b^low^).
(TIF)

S1 ARRIVE Checklist Checklist according to ARRIVE guidelines for reporting animal research *in vivo* experiments. (PDF)
