# Supplementary figures and images for: IL-4Rα-Dependent Alternative Activation of Macrophages Is Not Decisive for Mycobacterium tuberculosis Pathology and Bacterial Burden in Mice
Source: PLoS One. 2015 Mar 19;10(3):e0121070. doi: 10.1371/journal.pone.0121070 (PMC4366092; doi:10.1371/journal.pone.0121070)

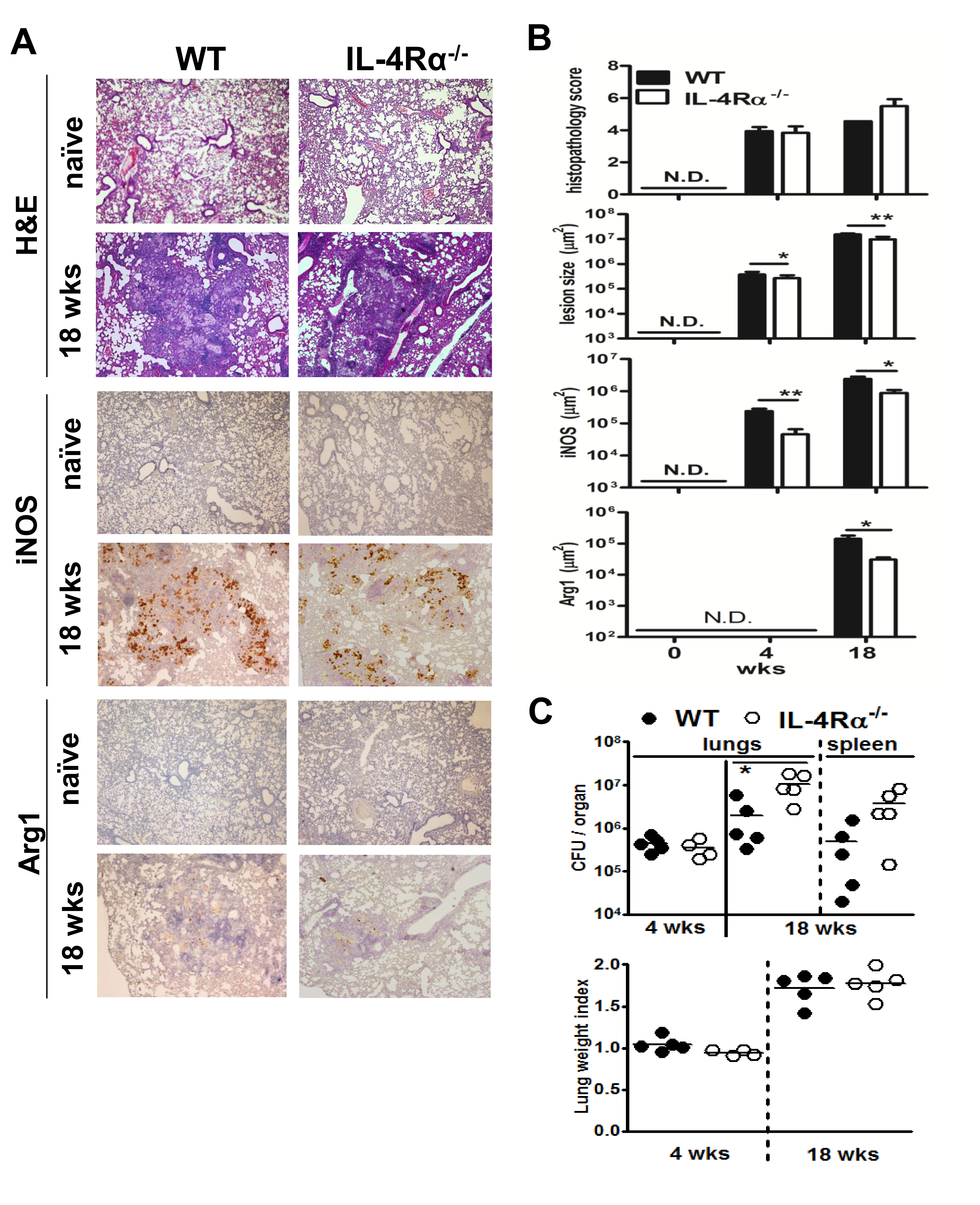

Supplement: S1 Fig — (A) H&E, iNOS and Arg1 staining from lung sections collected at 0 and 18 wks post infection, original magnification: 40X. (B) Histopathology score, quantification of lesion sizes, iNOS and Arg1 quantification. N.D. = not detectable. (C) Lung weight indexes and bacterial burden in the lungs and spleen are shown (5 mice/group, *P < 0.05). All data shown is representative of two independent experiments. (TIF) [file pone.0121070.s001.tif]

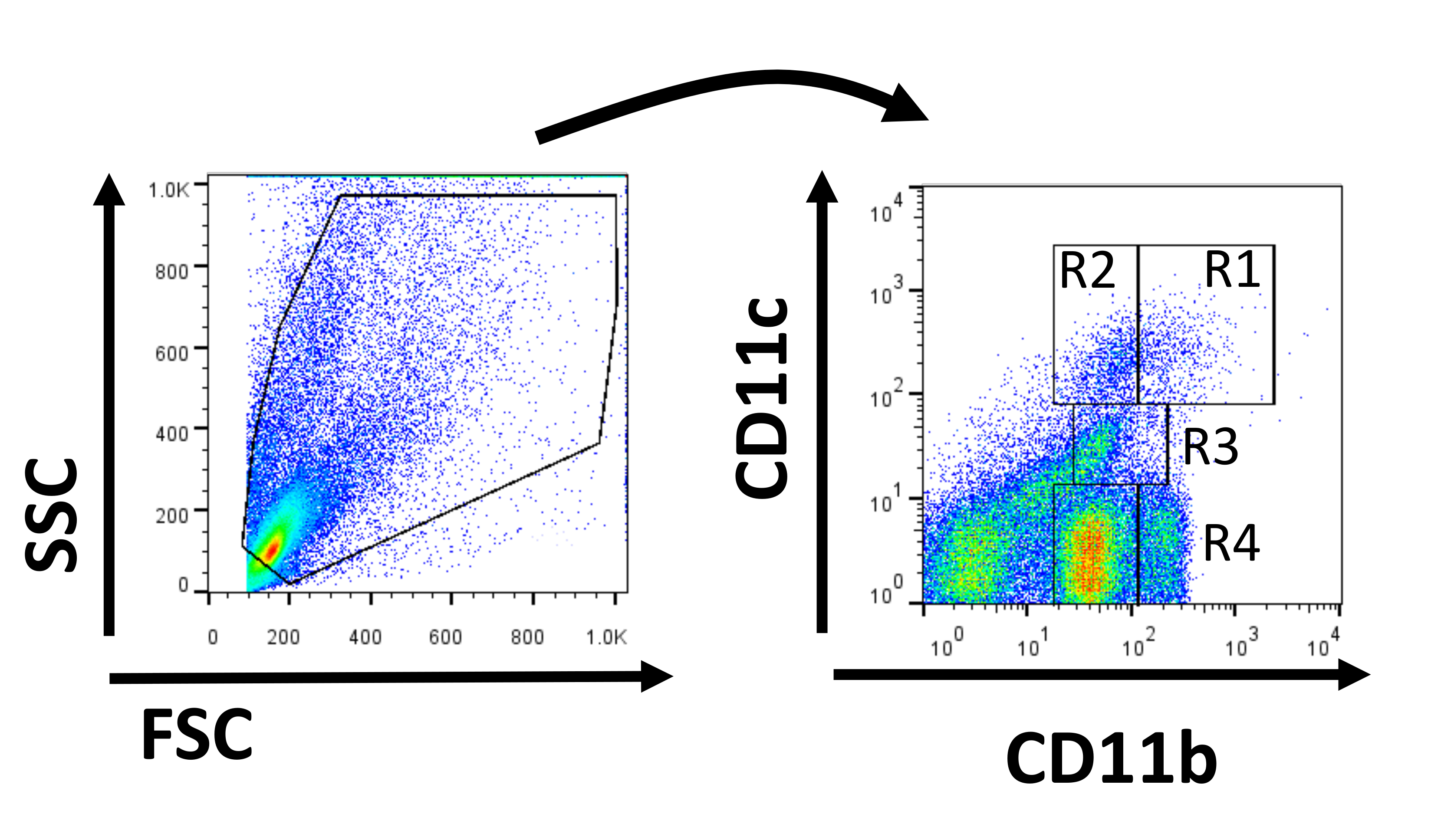

Supplement: S2 Fig — CD11b and CD11c-expressing subsets in the lung were defined as R1: conventional dendritic cells (CD11chighCD11bhigh), R2: alveolar macrophages (CD11chighCD11blow), R3: recruited interstitial macrophages (CD11clowCD11bmid) and R4: monocytes (CD11c-CD11blow). (TIF) [file pone.0121070.s002.tif]
